# Autoselection of Cytoplasmic Yeast Virus Like Elements Encoding Toxin/Antitoxin Systems Involves a Nuclear Barrier for Immunity Gene Expression

**DOI:** 10.1371/journal.pgen.1005005

**Published:** 2015-05-14

**Authors:** Alene Kast, Raphael Voges, Michael Schroth, Raffael Schaffrath, Roland Klassen, Friedhelm Meinhardt

**Affiliations:** 1 Institut für Molekulare Mikrobiologie und Biotechnologie, Westfälische Wilhelms-Universität Münster, Münster, Germany; 2 Fachgebiet Mikrobiologie, Universität Kassel, Kassel, Germany; Fred Hutchinson Cancer Research Center, UNITED STATES

## Abstract

Cytoplasmic virus like elements (VLEs) from *Kluyveromyces lactis* (Kl), *Pichia acaciae* (Pa) and *Debaryomyces robertsiae* (Dr) are extremely A/T-rich (>75%) and encode toxic anticodon nucleases (ACNases) along with specific immunity proteins. Here we show that nuclear, not cytoplasmic expression of either immunity gene (*PaORF4*, *KlORF3* or *DrORF5*) results in transcript fragmentation and is insufficient to establish immunity to the cognate ACNase. Since rapid amplification of 3' ends (RACE) as well as linker ligation of immunity transcripts expressed in the nucleus revealed polyadenylation to occur along with fragmentation, ORF-internal poly(A) site cleavage due to the high A/T content is likely to prevent functional expression of the immunity genes. Consistently, lowering the A/T content of *PaORF4* to 55% and *KlORF3* to 46% by gene synthesis entirely prevented transcript cleavage and permitted functional nuclear expression leading to full immunity against the respective ACNase toxin. Consistent with a specific adaptation of the immunity proteins to the cognate ACNases, cross-immunity to non-cognate ACNases is neither conferred by PaOrf4 nor KlOrf3. Thus, the high A/T content of cytoplasmic VLEs minimizes the potential of functional nuclear recruitment of VLE encoded genes, in particular those involved in autoselection of the VLEs via a toxin/antitoxin principle.

## Author Summary

The rather wide-spread and extremely A/T rich yeast virus like elements (VLEs, also termed linear plasmids) which encode toxic anticodon nucleases (ACNases) ensure autoselection in the cytoplasm by preventing functional nuclear capture of the cognate immunity genes, but how? When expressed in the nucleus, the mRNA of the VLE immunity genes is split into fragments to which poly(A) tails are added. Consistently, lowering the A/T content by gene synthesis prevented transcript cleavage and permitted functional nuclear expression providing full immunity against the respective ACNase toxin. Thus, internal poly(A) cleavage is likely to prevent functional nuclear immunity gene expression.

## Introduction

*Pichia acaciae* and *Kluyveromyces lactis* each contain two cytoplasmic virus-like elements (VLEs, also known as linear plasmids); i.e. pPac1-1 (12.6 kb), pPac1-2 (6.8 kb) and pGKL2 (13.5 kb), pGKL1 (8.9 kb) respectively [1,2]. The respective larger elements display substantial similarities to each other in terms of organization and gene content. They can exist without the smaller ones as they encode all proteins required for nucleus-independent cytoplasmic replication and maintenance [3]. The smaller VLEs pPac1-2 and pGKL1, respectively, which depend on the larger ones in terms of cytoplasmic transcription and/or replication, encode for the production of killer toxin complexes, zymocin (pGKL1) and PaT (pPac1-2) [reviewed in 4]. One subunit in either zymocin or PaT is highly conserved; it carries chitin binding and chitinase domains that recognize cell wall associated chitin of target cells as primary toxin receptor for subsequent import and/or activation [5,6,7]. In both zymocin and PaT, a rather hydrophobic stretch or subunit appears to manage membrane transfer of the cytotoxic subunits, PaOrf2 (encoded by pPac1-2 ORF2) and γ-toxin (encoded by pGKL1 ORF4). Although they hardly show any sequence similarity, they both act as anticodon nucleases (ACNases). The recently solved crystal structure of PaOrf2 revealed a unique fold, which shows no similarity to any known ribonuclease [8]. PaOrf2 specifically attacks tRNA^Gln^ *in vivo* and additionally cleaves *in vitro* tRNA^Glu^ and tRNA^Lys^ or synthetic stem-loop RNA derived from the tRNA^Gln^ sequence [8,9]. γ-toxin cleaves the same tRNAs *in vitro*, but *in vivo* its preferred target is tRNA^Glu^ [10,11]. While γ-toxin cleaves its target tRNA once at the 3`side of the wobble uridine, PaOrf2 apparently cleaves at the same position and additionally two nucleotides upstream, as judged from the appearance of two alternative cleavage products with full length tRNA from *S*. *cerevisiae* [9,10]. Since PaOrf2 but not γ-toxin evades a possible repair of the tRNA halves by cellular tRNA ligases, it was speculated that the presence of two cleavage sites might allow the excision of a di-nucleotide, rendering the target tRNA non-repairable [12,13,14,15].

VLE cured strains of *P*. *acaciae* and *K*. *lactis* are sensitive to their own respective toxins, proving that not only the killer phenotype but also the cognate immunity are encoded by the elements [1,2]. Indeed, PaT immunity is conferred by the only protein encoded by pPac1-2 (ORF4) that lacks a signal peptide for secretion [16] and immunity against zymocin had been postulated to be encoded by *KlORF3* of pGKL1 [17]. There is hardly any homology among *PaORF4* and *KlORF3*, and consistent with it, no cross-protection has been observed against zymocin or PaT [16]. PaT and zymocin are the most thoroughly studied VLE encoded ACNase killer toxins, but there are other systems in yeast [reviewed in 18], such as PiT from *Pichia inositovora*, a ribonuclease inducing specific fragmentation of 25S and 18S rRNAs [19], or DrT from *Debaryomyces robertsiae*, an ACNase resembling PaT and cleaving tRNA^Gln^ [20]. From an evolutionary point of view, toxin and immunity functions implemented in VLEs have to be considered as players of an autoselection system rather than providing advantages for the respective host [16,21], although the latter, clearly benefits from the conferred killer phenotype.

Intriguingly, *PaORF4* encoding PaT immunity could be heterologously functionally expressed solely from VLEs in the cytoplasm [16], i.e. when the gene was integrated into the pGKL-system of *Kluyveromyces lactis*. All efforts to express the immunity phenotype with *PaORF4* on nuclear episomal and centromeric vectors failed to establish self-protection against the ACNase toxin. Here, we show that *PaORF4* as well as the immunity genes from other VLEs (*KlORF3* and *DrORF5*) are nevertheless transcribed when the genes are governed by a yeast nuclear promoter in episomal vector backbones, but the mRNA becomes immediately fragmented thereby preventing translation that otherwise would yield a functional immunity protein. As exemplified for *PaORF4* and *KlORF3*, changing the primary structure from a rather high A/T bias to a much lower degree allowed for functional nuclear immunity expression, proving that the gene’s primary sequence information is sufficient to provide ACNase self-protection and that the native ORF context ensures autoselection of the VLE.

## Results

### VLE encoded immunity factors cannot be functionally expressed in the nucleus

**Fig 1:**
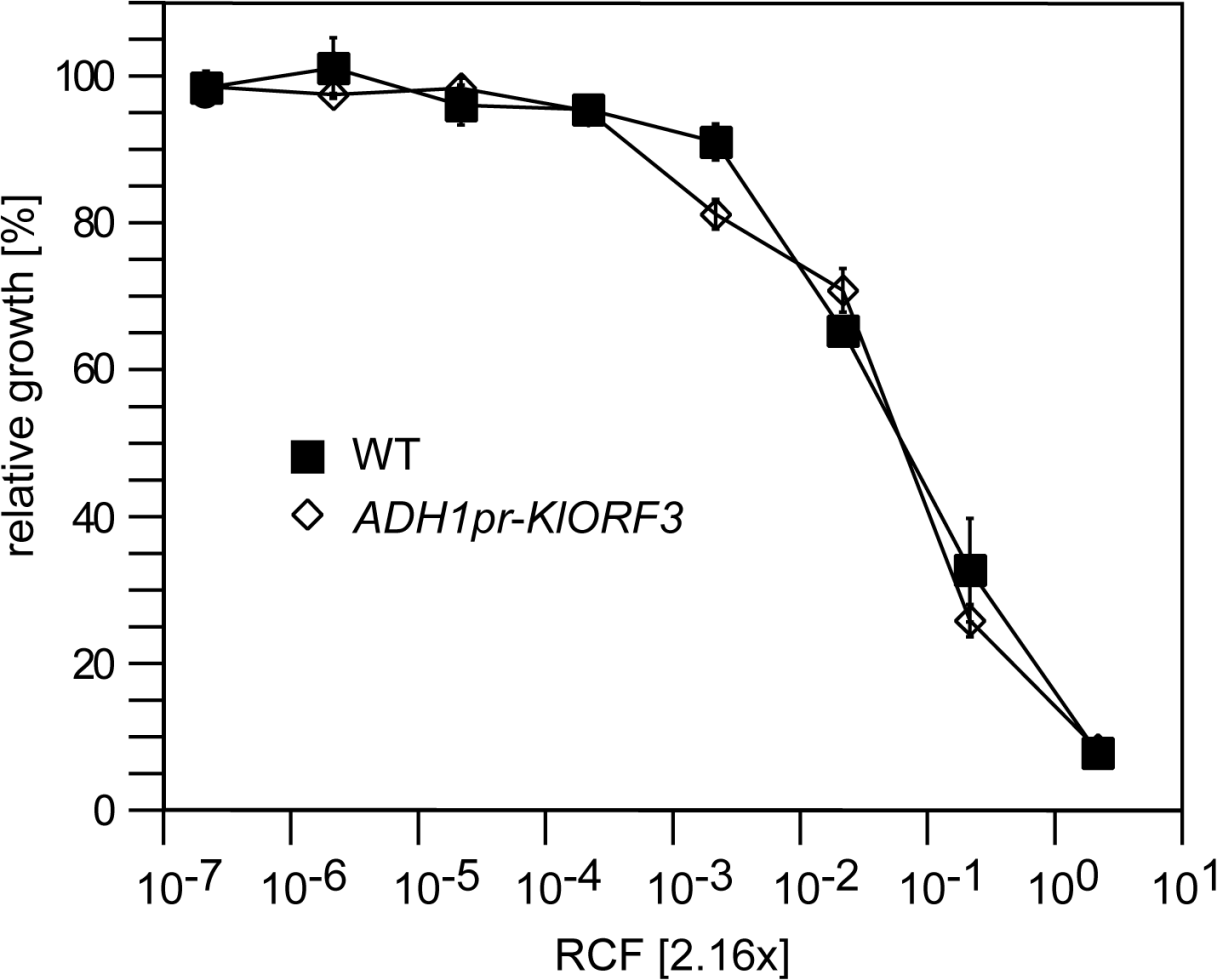
The zymocin immunity gene (*KlORF3*) does not confer immunity when expressed in the nucleus. *S*. *cerevisiae* strains without any vector (WT) or with YEKlO3 (*ADHpr-KlORF3*) were analyzed for zymocin resistance using the microtiter plate assay method. Relative growth was determined (OD_600nm_) after 24 hours at 30°C; values refer to strains grown in toxin-free medium. A relative concentration factor (RCF) of 1 relates to the toxin concentration in non-diluted supernatants. Each value represents a mean of triplicates.

For the three known VLE encoded ACNase toxin complexes PaT, zymocin and DrT, immunity functions were proposed to be encoded by *PaORF4*, *KlORF3* and *DrORF5*, respectively [16,17,20]. Subsequently, *PaORF4* and *DrORF5* were functionally expressed from their native promoters in the cytoplasm after integration of the genes into a VLE system (the pGKL1/2 system transferred to *S*. *cerevisiae*) [16,20,22]. Attempts to express both immunity genes in the nucleus after fusion of the ORFs to the constitutive *ADH1* promoter (*ADH1pr*), however, did not establish toxin immunity. This is in contrast to the putative zymocin immunity gene (*KlORF3*) which was previously identified on the basis of functional expression from the nucleus after fusion of the *KlORF3* to the *PGK* promoter [17]. Upon expression of the *PGKpr*-*KlORF3* construct, partial zymocin protection was observed in *S*. *cerevisiae* cells. However, the phenotype required the presence of the autonomous VLE pGKL2 while, nuclear expression in a standard *S*. *cerevisiae* strain devoid of any VLE did not confer detectable zymocin immunity [17]. To reconfirm this latter notion, we fused *KlORF3* to the alternative strong constitutive promoter *ADH1pr* and analyzed the zymocin response of the sensitive *S*. *cerevisiae* strain BY4741 containing the *ADH1pr*-*KlORF3* fusion in comparison to the wild type by the microdilution method. As shown in Fig 1, zymocin sensitivity indeed remains unaltered in the presence of the *ADH1pr*-*KlORF3* construct, supporting the conclusion that the VLE encoded immunity factors cannot be functionally expressed in the nucleus in a standard *S*. *cerevisiae* strain. In contrast, both *DrORF5* and *PaORF4* provide resistance to their cognate ACNase toxins (DrT and PaT) when expressed in the cytoplasm of a sensitive *S*. *cerevisiae* strain and in all assays conducted, complete, rather than partial immunity was observed [16,20].

### Northern analysis of immRNA reveals specific fragmentation in the nucleus

**Fig 2:**
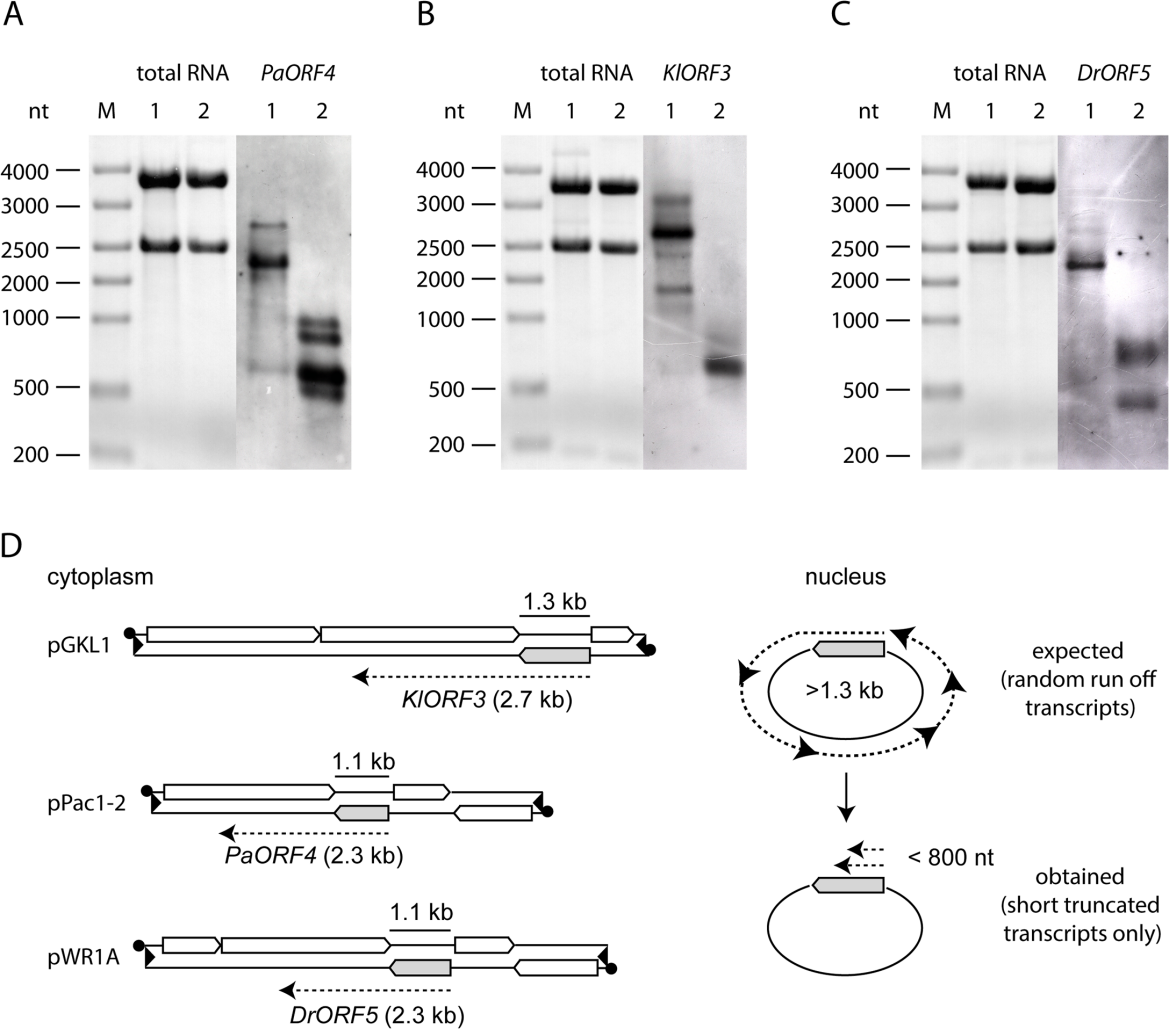
Northern blot analysis of immRNA from different genetic backgrounds. Total RNA isolated from strains expressing the respective immORFs from VLEs or nuclear vectors was probed using the respective immRNA specific DIG-labelled probes. A: *PaORF4* (1092 bp) expressed in *Pichia acaciae* from pPac1-1, pPac1-2 (1) or in *S*. *cerevisiae* 301 YEPaO4 (2). B: *KlORF3* (1287 bp) expressed in *K*. *lactis* from pGKL1, pGKL2 (1) or in *S*. *cerevisiae* CEN.PK2-1c YEKlO3 (2). C: *DrORF5* (1062 bp) expressed in *D*. *robertsiae* from pWR1A, pWR1B (1) or in *S*. *cerevisiae* CEN.PK2-1c YEDrO5 (2). D: Scheme of immORF transcription and mRNA sizes transcribed from cytoplasmic VLEs (pGKL1, pPac1-2 and pWR1B, left side) or from episomal vectors, which are located in the nucleus (right side). ImmORFs are grey shaded.

To analyze whether the observed failure of immunity expression from the nuclear vectors was due to a barrier in transcription or due to transcript instability, we analyzed the levels of mRNAs encoding immunity (immRNA) and their stability. ImmRNA from *S*. *cerevisiae* strains carrying nuclear fusions of the immunity factor encoding ORF (immORF) and the *ADH1pr* was compared with immRNA from natural expression hosts, where immORFs are expressed from the cytoplasm. In all cases, cytoplasmic expression yielded stable immRNA that exceeded the size of the corresponding full-length immORF (Fig 2). In contrast, nuclear expression of immORFs produced one (*KlORF3*), two (*DrORF5*) or four (*PaORF4*) distinct signal bands in Northern blots, which were significantly smaller in size than their corresponding full-length immORF (Fig 2). Thus, while immRNAs are stable, when expressed from their cognate VLEs in the cytoplasm, they are prone to fragmentation and get particularly instable when expressed in the nucleus. The lack of full length immRNA in the latter case is in line with the observed general lack of functional ACNase immunity when immORFs are expressed in the nucleus.

### Poly(A)-site processing leads to fragmentation of immRNA expressed in the nucleus

**Fig 3:**
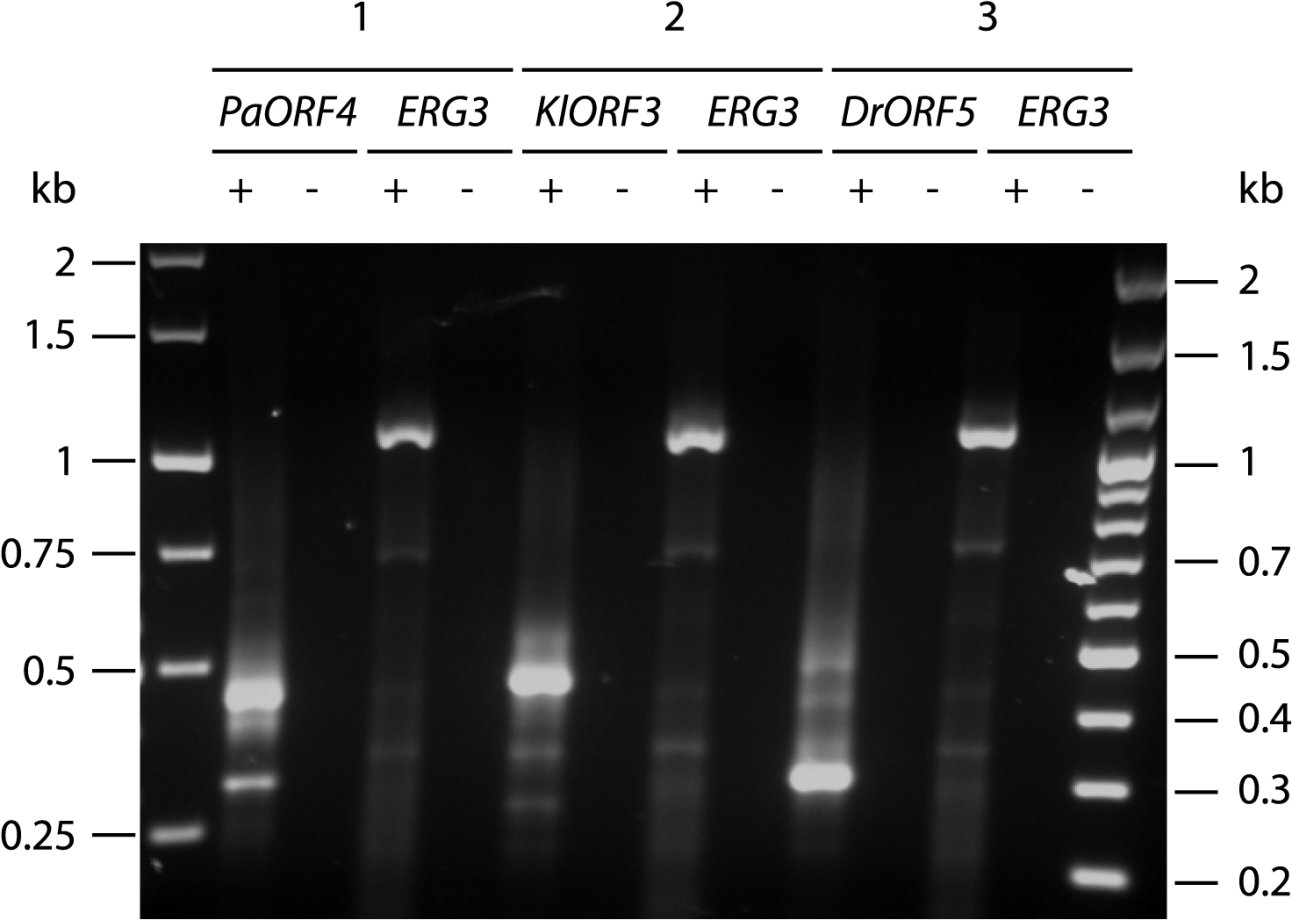
Amplification of immRNA fragments by 3´-RACE. Total RNA from *S*. *cerevisiae* CEN.PK2-1c strains carrying vectors YEPaO4 (1), YEKlO3 (2) or YEDrO5 (3) was transcribed to cDNA using a poly(A)-specific primer (+). Absence of DNA was checked by control reactions lacking the reverse transcriptase (-). ImmRNA was then amplified with one primer specific for the 5´ end of the immORF and a second primer complimentary to the poly(A)-specific anchor. *ERG3* cDNA was amplified using *ERG3* specific primers.

**Fig 4:**
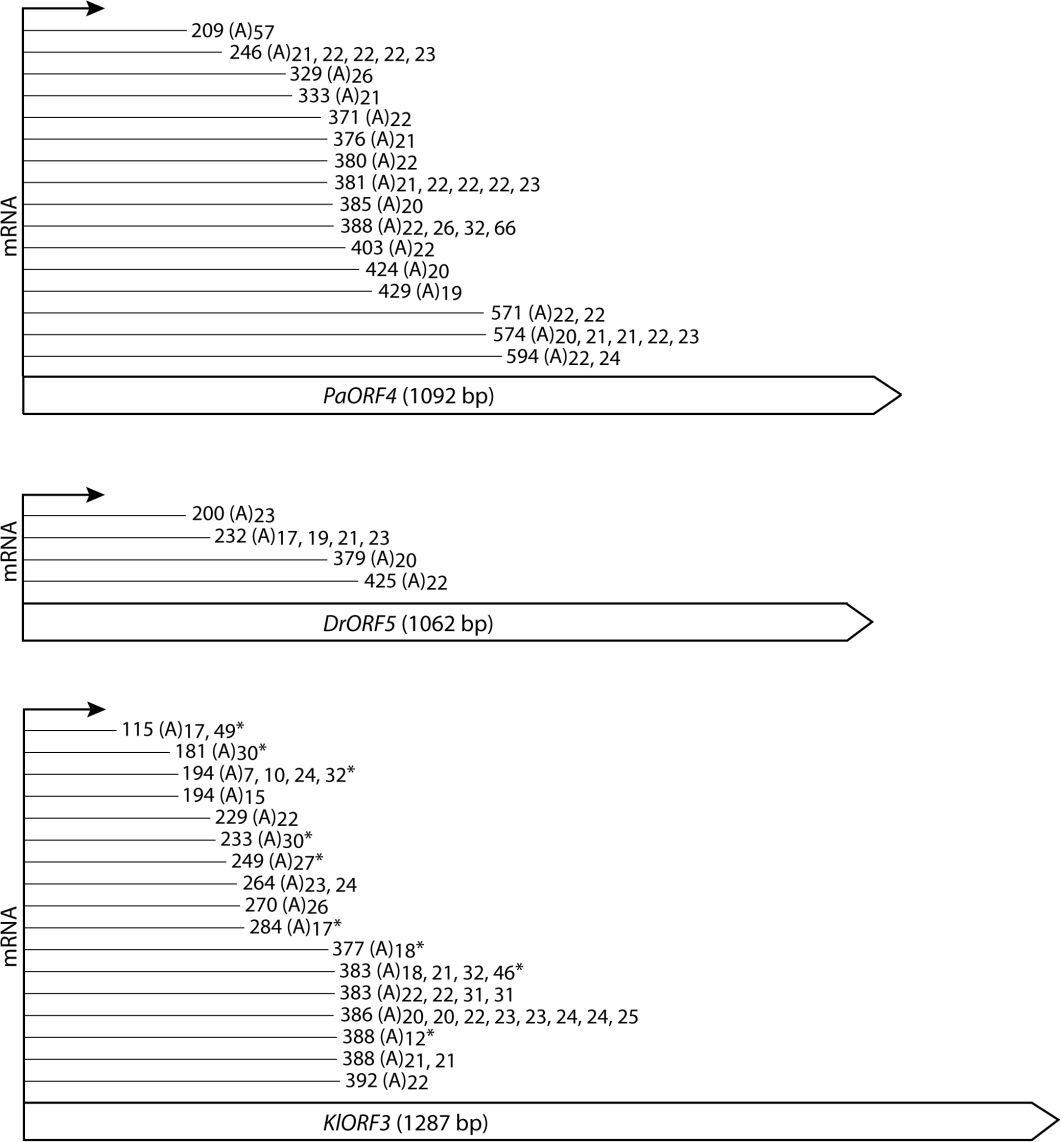
Schematic representation of the identified mRNA fragments of the immORFs *PaORF4* (A), *KlORF3* (B) and *DrORF5* (C). The cDNA fragments reached the indicated indicated nucleotide positions and were extended by a stretch of 7–66 adenyl (A) nucleotides, the length of which is indicated for each particular molecule that was obtained. Asterisks (*) mark fragments which were identified by the linker ligation method; all of the other fragments were identified by 3´-RACE. For complete gene sequences including the identified mRNA truncation sites we refer to S1 Fig

Since the poly(A) site processing machinery recognizes UA rich elements that appear to be quite diverse in *S*. *cerevisiae* [reviewed in 23], we explored the possibility that immRNA fragmentation of the highly A/T biased transcripts could be associated with recognition of random, internal poly(A) sites, leading to immORF fragmentation with the addition of poly(A) tails at the cleavage sites. We isolated total RNA from *S*. *cerevisiae* strains expressing *ADH1pr-KlORF3*, *ADH1pr-PaORF4* and *ADH1pr-DrORF5* and primed cDNA synthesis with a poly(A)-specific oligonucleotide. Such cDNAs were analyzed by PCR using immORF specific oligonucleotides, binding at the 5’ end together with an oligonucleotide complementary to the poly(A)-specific anchor. As a control, the *ERG3* mRNA was amplified from the same cDNA preparations. For all immORFs, several 3’ RACE products were obtained, all of which were smaller than the minimum expected gene size (Fig 3), suggesting the presence of poly(A) stretches at the 3´ ends of the immRNA fragments. Such result agrees with the specific fragmentation of the nuclearly expressed immRNAs followed by the addition of poly(A) tails. Fragments of each PCR reaction were extracted from the gel and cloned; sequencing identified the fragments to perfectly match the 5`terminal regions of the respective immRNAs, which are truncated at their 3´ends and extended by attachment of several (16 to 66) adenines (Fig 4).

To ensure that the data obtained from 3’RACE experiments did not result from unspecific internal priming within A-rich mRNA regions, a linker ligation method was applied to exemplarily identify the immRNA ends of *KlORF3*. The linked *KlORF3* fragments were amplified using a gene specific and a linker specific primer and after cloning analyzed by sequencing. All identified fragments contained a poly(A) tail consisting of 7–49 adenyl nucleotides. The results confirm the previous observations. Both methods identified that cleavage and polyadenylation of each of the immRNAs happens not only at one definite, but at multiple positions. For example, among the 33 sequenced *PaORF4* mRNA fragments, 15 different polyadenylation sites in a region between positions 209 nt and 594 nt were mapped (Fig 4 and S1 Fig). The identified positions in the mRNA truncation products and their frequency of occurrence for each immRNA are summarized in Fig 4.

### Lowering immORF A/T content enables functional nuclear expression

**Fig 5:**
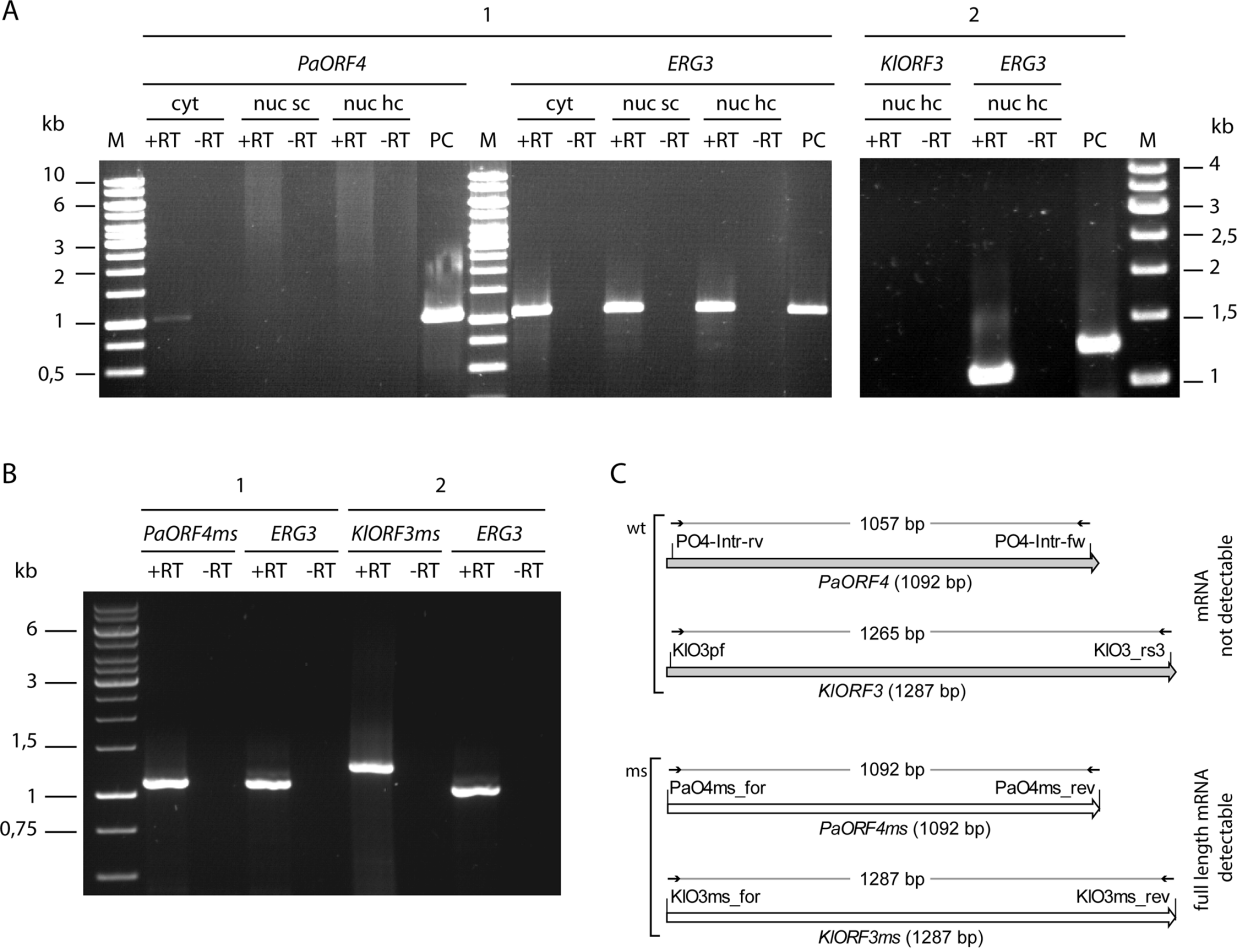
RT-PCR analysis of immRNA. mRNA of *PaORF4*/*PaORF4ms* and *KlORF3*/*KlORF3ms* or the control gene *ERG3* was analysed by reverse transcription (+RT) using total RNA as the template. The absence of contaminating DNA was checked by control reactions lacking the reverse transcriptase (-RT). A: mRNA of the original A/T-rich immORFs *PaORF4* and *KlORF3* was analysed using total RNA isolated from *S*. *cerevisiae* CEN.PK2-1c strains expressing *ADH1pr*-*PaORF4* (1) or *ADH1pr*-*KlORF3* (2) from nuclear single copy vectors (nuc sc) or nuclear high copy vectors (nuc hc) or from *S*. *cerevisiae* F102.2 MS1607 expressing *PaORF4* from a cytoplasmic VLE (cyt). PC: as a positive control for checking primer binding vector DNA carrying the immunity genes *PaORF4* or *KlORF3* was used. B: Reverse transcription of mRNA of the codon adjusted, A/T-decreased immunity genes *PaORF4ms* and *KlORF3ms* using as the template total RNA isolated from strains expressing *ADH1pr*-*PaORF4ms* (1) or *ADH1pr*-*KlORF3ms* (2) from high copy vectors. C: Schematic representation of RT-PCR analyses for the detection of immRNAs. Primers, depicted as small arrows, are denoted as such (for sequences see S3 Table). wt denotes the original immunity gene sequences (grey shaded large arrows), ms denotes the modified (G/C rich) sequences (white large arrows). The expected sizes of RT-PCR products refer to the size of the structural genes and are given in base pairs (bp).

Since the above results suggested the possibility that the high A/T content of immRNAs limits their nuclear expression due to ORF-internal poly(A) site processing, we analysed whether a reduction of the A/T content of *PaORF4* and *KlORF3* improves their expression from the nucleus. Synthetic variants of both genes were generated, where most of the A/U rich codons were replaced by synonymous more G/C rich codons via gene synthesis (S2 Fig). As a result, the G/C content for *PaORF4* increased from 21% to 45% in the synthetic variant *PaORF4ms* and *KlORF3* increased in G/C content from 22% to 54% in the synthetic variant *KlORF3ms* without altering the amino acid sequence. Both variants were cloned into the same vector backbone previously used to study the native, unchanged genes, resulting in a set of multicopy plasmids carrying immORF fusions to the *ADH1* promoter, where immORFs remain either unchanged in codon usage (78–79% A/T) or exhibit a significantly reduced A/T content (46–55%). All constructs were expressed in a PaT or zymocin sensitive VLE-free *S*. *cerevisiae* strain and the presence of full length immRNA was comparatively analysed by RT-PCR (Fig 5). As a control, the *ERG3* mRNA was detected in all strains in parallel. Full length immRNA was generally absent in the strains expressing the A/T-rich native (non-modified) versions of the immORFs (Fig 5A), which is in agreement with the results obtained by Northern analysis (Fig 2). In striking contrast, however, full length immRNA becomes detectable when low A/T content codon usage variants *PaORF4ms* and *KlORF3ms* are expressed from the same nuclear constructs (Fig 5B). Thus, lowering the A/T content clearly improves immORF expression in the nucleus compared to the natural gene variants being expressible only in the cytoplasm.

**Fig 6:**
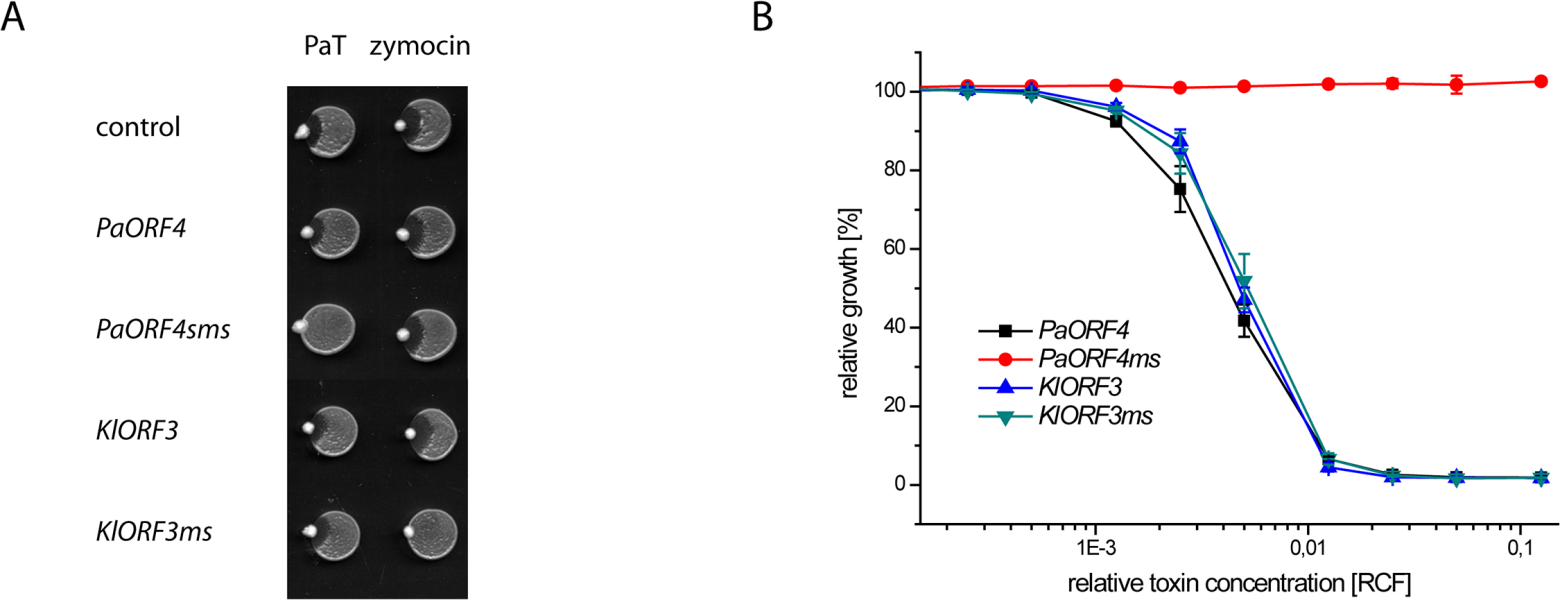
The codon adapted immORFs *PaORF4ms* and *KlORF3ms* confer resistance to the respective toxins PaT and zymocin. A: *S*. *cerevisiae* CEN.PK2-1c transformed with yeast episomal vectors carrying *ADH1pr* fusions of either the A/T-rich (*PaORF4* and *KlORF3*) or the A/T-decreased (*PaORF4ms* and *KlORF3ms*) immunity genes or the empty vector (control) were dropped on YPD agar, inoculated with *P*. *acaciae* (PaT) or *K*. *lactis* (zymocin) at the rim and incubated over night at 30°C. B: Microtiter plate assays. Supernatants from a *P*. *acaciae* culture were added in different concentrations (RCF) to micro cultures of the yeast strains described in A. Relative growth was determined (OD_600nm_) after 24 hours at 30°C; values refer to strains grown in toxin-free medium. A relative concentration factor (RCF) of 1 relates to the toxin concentration in non diluted supernatants. Each value represents a mean of triplicates.

To check whether such improvement also enables functional immORF expression, ACNase immunity of strains carrying the different immORF expression constructs was scored by the eclipse plate and microdilution assays (Fig 6). Consistent with previous results, expression of A/T-rich versions of *PaORF4* or *KlORF3* did not confer a detectable immunity phenotype to the PaT or zymocin producers, respectively. When the low A/T-content variants *PaORF4ms* or *KlORF3ms* were expressed, however, sensitivity to the cognate ACNase toxin producer was entirely lost. At the same time, the *PaORF4ms* expressing strain remained sensitive to the zymocin producer (Fig 6A and Fig 6B) and the *KlORF3ms* expressing strain remained sensitive to the PaT producer (Fig 6A). These results indicate that although lowering the A/T contents in two functionally distinct immORFs suffices to overcome the observed nuclear expression barrier, the immunity factors, once being expressed, do not confer ACNase cross-protection to non-self yeast strains.

### *KlORF3ms* confers full, pGKL2 independent immunity to intracellular γ-toxin

**Fig 7:**
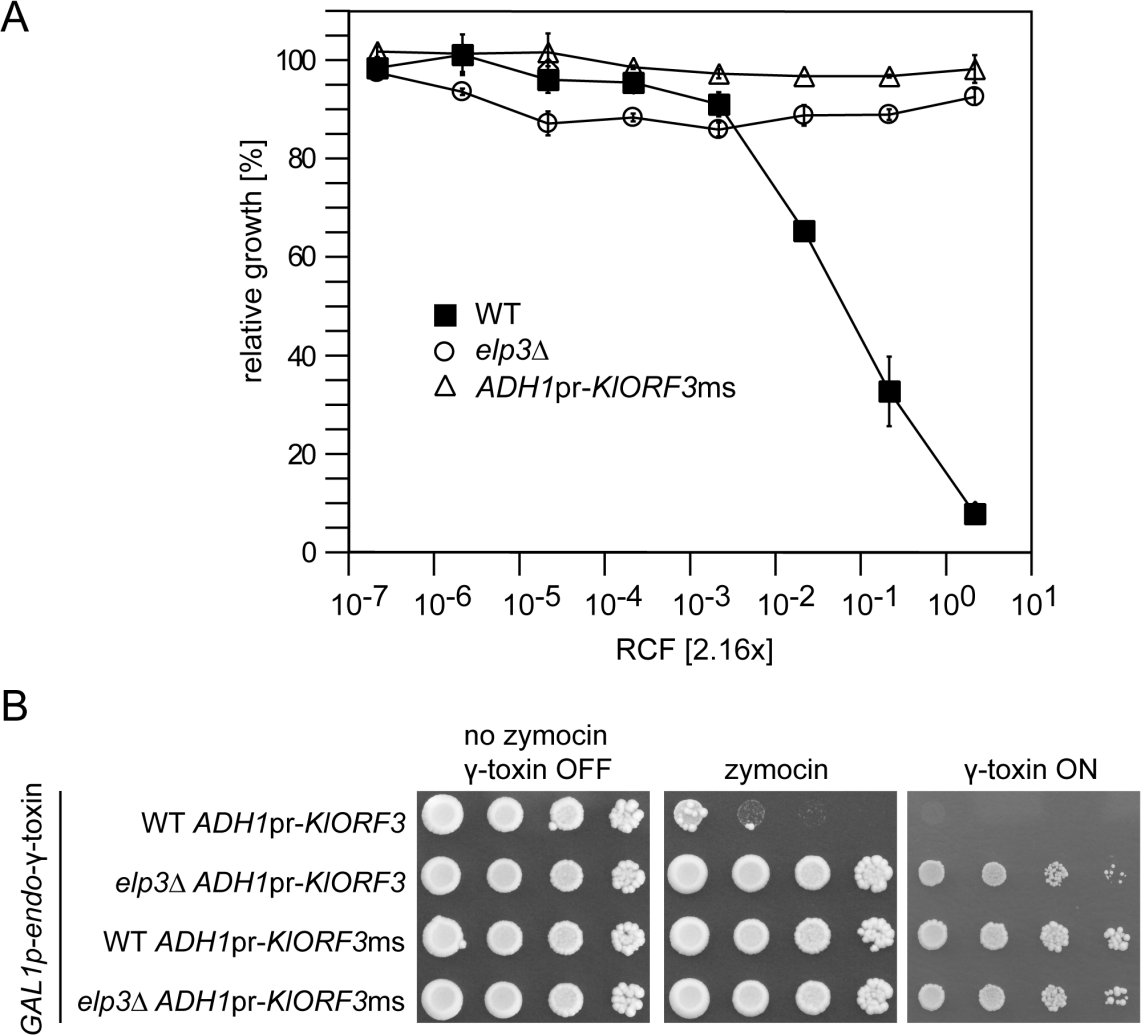
The zymocin immORF confers full immunity to exo zymocin and intracellularly expressed ACNase subunit γ-toxin. (A) Exo-zymocin resistance of isogenic WT, *elp3*∆, and WT strains carrying YEKlO3ms (*ADHpr-KlORF3ms*) analyzed by microtiter plate assay as described in Fig 1. (B) Intracellular coexpression of zymocin immORF and γ-toxin. Tenfold serial dilutions of cultures were spotted on YPD media (no zymocin, γ-toxin OFF and zymocin), YPD media supplemented with zymocin (zymocin) or YNB media with galactose as the sole carbon source (γ-toxin ON). All strains analyzed in (B) carry pRK57 for GAL driven, intracellular expression of γ-toxin and additionally carry YEKlO3 (*ADH1pr-KlORF3*) or YEKlO3ms (*ADH1pr-KlORF3ms*). WT and *elp3*∆ indicate the additional presence or absence of a deletion in *ELP3* (*elp3*∆).

Our *KlORF3ms* expression studies (Fig 6A) confirm the previous proposal by Tokunaga *et al*., [17] that pGKL1 encoded Orf3 protein confers zymocin immunity. As *KlORF3ms* yields protection to the zymocin producer *K*. *lactis* in the background of *S*. *cerevisiae* strain BY4741, there is evidently no general requirement for the presence of the pGKL2 VLE to establish immunity. In previous experiments, the immunity phenotype associated with the *PGKpr*-*KlORF3* construct and the pGKL2 VLE was only partial, since exogenous purified zymocin induced detectable growth inhibition [17]. To check whether the pGKL2 independent immunity conferred by nuclear expression of *ADH1pr*-*KlORF3ms* also is partial, we analyzed the zymocin response using the microdilution assay. We compared *KlORF3ms*-induced zymocin protection in WT cells with *elp3* cells not carrying any immORF. The latter condition prevents zymocin induced tRNA cleavage due to absence of the crucial wobble uridine mcm^5^s^2^-modification and confers full toxin resistance [10,11]. We observed no difference between the zymocin response of *elp3* cells not expressing any immORF and *ELP3* cells expressing *KlORF3ms*; only *ELP3* wild type cells without *KlORF3ms* showed sensitivity to zymocin (Fig 7A). To further check the dominant nature of *KlORF3ms* induced immunity and to analyze whether the KlOrf3 protein is capable of intracellular inactivation of γ-toxin, as suggested in earlier work [17], we constructed strains co-expressing *KlORF3*/*KlORF3ms* and *GAL1pr*-driven, multi copy *KlORF4* devoid of its signal peptide encoding region, leading to intracellular accumulation of the ACNase subunit γ-toxin (KlOrf4). As a control, both immORF constructs were introduced into the *elp3* strain, where the need for an immORF is overcome by preventing tRNA cleavage in the first place. Galactose induced expression of the γ-subunit proved inhibitory to the strain expressing the A/T-rich variant *KlORF3* only; as expected, the *elp3* mutation prevented toxic effects of γ-toxin but also *KlORF3ms* entirely prevented growth inhibition by intracellular γ-toxin and the additional removal of *ELP3* did not improve growth of the strain under inducing conditions (Fig 7B). Thus, zymocin immunity acts, similar to the previously studied PaT and DrT immunity functions at the intracellular stage and provides true immunity rather than partial resistance, independent of any pGKL2 encoded functions. Since we detected no cross resistance of *KlORF3ms* expressing strains to the non-cognate ACNase toxin PaT (Fig 6A and Fig 6B) and *PaORF4ms* did not protect detectably from zymocin (Fig 6A), the two immunity proteins PaOrf4 and KlOrf3 are highly specific for each of their cognate ACNase subunits.

## Discussion

PaT, DrT and zymocin are the three known examples of eukaryotic protein toxins with ACNase activity. All three are encoded by non-autonomous VLEs (pPac1-2; pWR1A and pGKL1) persisting in the cytoplasm of different yeast species. Crucial functions for cytoplasmic transcription and DNA replication, processes normally occurring in the nucleus, are supplied in each case by a larger VLE (pPac1-1; pWR1B and pGKL2). Among these are a uniquely structured RNA polymerase [24] as well as a virus like mRNA capping enzyme [25,26] to generate capped, cytoplasmic mRNAs from unique cytoplasmic promoters [27,28,29]. Generally, VLE genes cannot be expressed in the nucleus due to non-recognition of the cytoplasmic promoters by the nuclear (host encoded) RNA polymerases. The presence of genes on pPac1-2 and pWR1A mediating immunity against PaT and DrT was previously shown by integration into the pGKL1/2 system transferred to *S*. *cerevisiae*. The parental pGKL1/2 carrying *S*. *cerevisiae* strain produces zymocin and its cognate immunity factor but was sensitive to DrT and PaT. This sensitivity was entirely lost upon integration of *DrORF5* and *PaORF4*, respectively [16,20]. Since both, DrOrf5/PaOrf4 and DrOrf3/PaOrf2 display detectable sequence homology and there is significant DrT/PaT cross protection mediated by PaOrf4, a direct recognition of the matching (PaOrf2) or nearly matching (DrOrf3) ACNase by the immunity factor was suggested. In support, PaOrf4 can disable toxic *in vivo* effects of intracellular PaOrf2 and both proteins were shown to form a complex *in vitro* that inhibits the ACNase activity of PaOrf2, resembling the mode of action of tRNase colicin immunity factors, which tightly bind and occlude the tRNase active site [8,30]. Similarly, *in vivo* studies with DrOrf5 showed that it protects against the *in vivo* tRNase activity of the intracellular DrOrf3 subunit [20], hinting at a similar immunity principle as for PaOrf4. Co-crystal structures of VLE-encoded tRNases with their cognate immunity proteins will be required to determine whether immunity factors against toxic tRNases as evolutionary diverse as prokarytotic colicins and VLE encoded killer toxins indeed share a similar mechanistic strategy to bind and occlude the tRNase active site.

For zymocin, understanding of the immunity factor function had been less advanced; published data [17] indicated an as yet undefined requirement for the VLE pGKL2 to establish KlOrf3 mediated immunity and in contrast to PaT and DrT immunity functions, the zymocin immunity factor appeared to provide partial protection only. Additionally, it was suggested that the zymocin immunity factor protects from intracellular γ-toxin based on the observation that pGKL1/2 carrying cells are resistant to galactose-induced expression of a signalpeptide-less *KlORF4* gene [17], but no similar protection has been shown for the isolated *KlORF3* gene. Since heterologous expression of *KlORF3* precluded the use of the cytoplasmic pGKL1/2 based expression system, we encountered the general problem that even the established immunity genes *PaORF4* and *DrORF5* could not be expressed in the nucleus after replacement of the cytoplasmic promoter by well characterized nuclear promoters. As the same outcome was observed for the zymocin immunity gene, a general principle inhibitory to nuclear expression of these immunity genes became obvious. Since AT rich immunity genes are efficiently translated by the host’s translational machinery when the corresponding mRNA is generated in the cytoplasm by a VLE encoded transcriptional machinery but not when the same mRNA is generated in the nucleus, a nuclear transcriptional rather than a cytoplasmic translational barrier appeared to exist. Northern, RACE and linker ligation analysis now shows that nuclear expression generally ends up in fragmentation of the immRNAs which goes along with the addition of poly(A) tails.

Poly(A) site recognition is thought to basically involve the presence of the AAUAAA poly(A) signal (PAS), together with a GU-rich sequence as a downstream element [reviewed in 23]. However, unlike higher eukaryotes, yeast apparently tolerates a high degree of variation in individual poly(A) site recognition elements, as was derived from the analysis of expressed sequence tags generated by oligo(dT) primed cDNA synthesis [31]. For example, the PAS element in yeast is simply characterized by being A-rich. Thus, extremely AU-rich transcripts, such as VLE derived genes exhibit a high probability of ORF internal poly(A) site recognition and processing when moved to the nucleus. In support of this, we show that lowering the A/T content by defined gene synthesis is sufficient to prevent immRNA fragmentation in the nucleus allowing for functional expression of PaT and zymocin immunity phenotypes.

Our analysis with the synthetic *KlORF3ms* construct shows that the zymocin immunity protein indeed acts intracellularly and provides true self-protection rather than partial resistance. Thus, all eukaryotic ACNase immunity proteins may, like PaOrf4, recognize and inhibit their cognate ACNase. In support of a specific recognition, cross immunity against DrT but not zymocin can be provided by PaOrf4, whereas KlOrf3 provides immunity solely and specifically to zymocin. Since ACNase subunits of DrT and PaT are detectably similar but no similarity exists between DrT/PaT and zymocin, such similarity/non-similarity is apparently recognized by the immunity proteins. The initial detection of partial zymocin resistance in a strain carrying *PGKpr*-*KlORF3* in the nucleus as well as pGKL2 in the cytoplasm [17] may be correlated to the fact that the fusion of *KlORF3* to the *PGKpr* did not eliminate the upstream conserved sequence (UCS) element of *KlORF3*. Since UCS sequences have been shown to be sufficient for mediating cytoplasmic transcription [28] and toxin resistance was only seen when pGKL2, i.e. the UCS-recognizing RNA polymerase was present, it appears possible, that partial zymocin immunity was due to cytoplasmic transcription of the *PGKpr*-*KlORF3* construct, which may have been located transiently in the cytoplasm after transformation. Such transient cytoplasmic availability may constitute the basis for the observed partial zymocin resistance as opposed to full immunity in this study.

Only rather recently nuclear sequences of plasmid and viral origin (NUPAVs) were detected which—eponymously—result from evolutionary capture of plasmid and VLE-genes by the yeast nucleus [32]. Indeed, cytoplasmic VLE based genes can frequently and repeatedly be trapped by the nucleus, as explicitly shown for pDH1A from *Debaryomyces hansenii*, which represents the most recent ancestor of NUPAVs so far known [33]. Taken also into consideration that some ORFs of VLEs, such as the toxin genes have been cloned and successfully expressed from nuclear vectors [19,34,35] the risk is immediately imposed on a VLE system that upon nuclear immunity gene capture autoselection is disabled, which is yet mandatory for VLE long term propagation. While chromosomally encoded yeast killer toxins are traditionally considered as factors beneficial to the producer cell due to the ability to eliminate competitors, VLE encoded toxins additionally or even predominantly serve to counterselect for spontaneous plasmid free segregants, clearly resembling the autoselective properties of bacterial toxin/antitoxin systems [16,21,36]. In *Pichia acaciae*, *Kluyveromyces lactis* and *Debaryomyces robersiae* toxin encoding cytoplasmic VLEs can be easily eliminated under laboratory conditions, which in all cases generates toxin sensitive segregants. Such situation differs from the vast majority of chromosomally encoded toxins, which are routinely not active against the producing species which supports that VLE encoded killer toxins function to kill spontaneous VLE-free segregants. However, such function does not exclude additional benefits to producer cells that are provided by the ability to kill other yeasts in a given environment. Since VLE-derived NUPAVs in different stages of degeneration can be detected in various yeast genomes [32,33], the high A/T content of VLEs in general may serve to minimize the potential for domestication of VLE based genes by the host, which might be particularly relevant for the immunity function that, if domesticated and separated from a toxin encoding VLE, would eliminate the positive selective pressure incurred by the toxin on the maintenance of the VLE system. In other words, spontaneous VLE-free segregants would no longer be eliminated in an environment of VLE containing, toxin secreting sister cells. Interestingly, among the VLE derived NUPAVs described by Frank and Wolfe, 2009, immunity genes constitute the largest group. We identified an additional immunity derived NUPAV in the yeast *Pichia sorbitophila* [37], which appears to be almost intact and closely related to the *DrORF5*/*PaORF4* genes (S3 Fig). The gene (Piso0_001880) located close to the end of chromosome F spans 729 bp and contains at its 5’end an extended region of similarity to the immORFs where, however, the ATG and the VLE promoter appear to be lost, leading to the annotation of an internal ATG as the gene’s startcodon. Importantly, Piso0_001880 has an A/T content of 75.5% which resembles the typical VLE characteristics and differs significantly from *P*. *sorbitophila* genome average (58.6%). We assume that Pios0_001880 represents a rather recent VLE derived domestication in an early stage of degeneration that may be related to the nuclear expression barrier caused by extreme A/T content. In support, no entirely intact immunity-NUPAV has been identified so far, suggesting a general incompatibility of A/T rich VLE genes with the nuclear transcript processing machinery.

## Materials and Methods

### Strains, media and general methods

The cloning host *Escherichia coli* DH5αF’ was grown in Luria-Bertani (LB) medium (0.5% yeast extract, 1% peptone, 0.5% NaCl) supplemented with ampicillin (100 μg ml^-1^) at 37°C. Yeast strains used in this study are listed in S1 Table. They were grown either in YEPD medium (1% yeast extract, 2% peptone, 2% glucose) or in yeast nitrogen base (Difco, Detroit, MI, USA) at 30°C. Transformation of *S*. *cerevisiae* was performed according to the PEG/lithium acetate method [38].

### Construction of plasmids

Plasmids used in this study are listed in S2 Table. The immORFs were amplified by PCR using total DNA of the killer yeasts *P*. *acaciae*, *D*. *robertsiae*, *K*. *lactis* as template and the primers listed in S3 Table (*PaORF4*: PO4-NdeI-rv and PO4-fw, *KlORF3*: KlO3rev_NdeI and KlO3for, *DrORF5*: DrO5rev_NdeI and DrO5for). The PCR products were blunt-end cloned into *Eco*RV restricted pSK- plasmid to yield pSKPaO4, pSKKlO3 and pSKDrO5. The pSKDrO5 plasmid was modified by site-directed mutagenesis to remove a *DrORF5*-internal *Nde*I restriction site using the primers mut_NdeI_for and mut_NdeI_rev. The A/T decreased gene versions *PaORF4ms* and *KlORF3ms* were synthesized by GeneArt (Regensburg, Germany) and delivered in a vector containing *Nde*I and *Hin*dIII restriction sites upstream and downstream of the respective ORF. All ORFs were released from their vectors via *Nde*I and *Hin*dIII and cloned into a likewise restricted pSKpADH1. The *ADH1pr*-immORF fusions were then ligated into the 2μ vector YEplac195 using the restriction sites *Kpn*I and *Sac*I (YEPaO4, YCPaO4 YEKlO3, YEDrO5, YEPaO4ms) or *Sma*I and *Hin*dIII (YEKlO3ms). For coexpression of *KlORF3*ms and γ-toxin, the *Eco*RI-*Bgl*II insert of pABY1643 (*GAL1*pr-γ-toxin-GST; [10]) was subcloned into *Eco*RI-*Bam*HI digested YEplac181.

### Killer toxin assays

For the microtiter plate assay the partially purified toxins PaT and zymocin were obtained from culture supernatants of *P*. *acaciae* NRRL Y-18665 and *K*. *lactis* AWJ137 by ultrafiltration as described previously [20]. Different toxin concentrations were applied in microtiter plates as described in Klassen *et al*., [39]. Cell growth was monitored photometrically in a Multiscan FC Microplate Photometer (Thermo Fisher Scientific, Waltham, MA, USA) at 620 nm.

To check the sensitivity of a strain in the eclipse assay, a drop of 7 μl of the cell suspension was spotted on a YEPD agar plate. The killer strain was then placed at the rim of the drop and the plate was incubated at 30°C overnight.

Effects of intracellularly expressed, galactose-inducible toxin subunits on the growth of certain strains were checked with the drop dilution assay. Cultures were serially diluted and 5 μl aliquots were spotted on YNB medium containing glucose (repressing condition) or galactose (inducing condition) and incubated for several days at 30°C. Zymocin containing YPD plates were prepared by spreading 300 μl of filter sterilized, concentrated supernatant (RCF 5) of *K*. *lactis* AWJ137.

### Northern analysis

Total RNA of different immunity ORF expressing yeast strains was isolated after over night cultivation in YEPD medium. 1.5 μg of each RNA sample were separated by a denaturating 1.5% agarose gel electrophoresis (20 mM MOPS, 8 mM sodium acetate, 1 mM EDTA, 0.74% formaldehyde, pH 7.0) and blotted onto a positively charged nylon membrane (Roche Diagnostic GmbH, Mannheim, Germany). The blotting success and RNA integrity were controlled by methylene blue staining (0.02% methylene blue, 0.3 M sodium acetate, pH 5.2). Hybridization was performed at 57–61°C overnight in hybridization buffer containing 50% formamid and a DIG-labelled RNA probe specific for the mRNAs of *PaORF4*, *KlORF3* and *DrORF5*, respectively. Probes were prepared by amplifying the gene sequences using the primers (PaORF4_probe_for/PaORF4_probe_revT7, KlO3proberevT7/KlO3res1, DrO5_probe_rev_T7/DrO5_rs1, S3 Table) and labelled with the DIG RNA Labeling Kit (SP6/T7) (Roche Diagnostic GmbH, Mannheim, Germany) according to the manufacturer’s instructions. For detection a phosphatase-conjuncted anti-DIG antibody and a chemilumiscent alkaline phosphatase substrate CDPstar (Roche Diagnostic GmbH, Mannheim, Germany) were applied, and signals were visualized by exposure to X-Ray films.

### cDNA synthesis

For cDNA synthesis the RevertAid H minus first strand cDNA synthesis kit (Fermentas, St. Leon-Rot, Germany) was applied according to the manufacturer’s instructions. All primers used for cDNA synthesis are listed in S3 Table.

### Isolation of total RNA

Total RNA was isolated as previously described (Klassen et al., 2008) or, alternatively, by making use of the RNeasy Mini Kit (Qiagen, Hilden, Germany) according to the manufacturer’s instructions.

### mRNA detection by RT-PCR analysis

cDNA was synthesized using random hexamer primers and total RNA as template. 1 μl of cDNA was used as the template for PCR reactions applying the Phusion High fidelity DNA polymerase (Thermo Fisher Scientific) and primer combinations PaO4intr-fw/PaO4intr-rv (*PaORF4*), PaO4ms_for/PaO4ms_rev (*PaORF4ms*), KlO3_rs3/KlO3pf (*KlORF3*) and KlO3ms_rev/KlO3ms_for (KlORF3ms) (for primer binding positions see Fig 5C).

### Identification of immRNA 3´ ends (3´ RACE and linker ligation)

Primer design for 3´ RACE experiments was based on the protocol of Scotto-Lavino *et al*., [40]. Polyadenylated RNA was transcribed to cDNA using the poly(A) complimentary primer QT22. The cDNA was used as template for PCR with primer Q0 (binds to a QT22 anchor) and an immORF specific primer at an annealing temperature of 58°C. PCR products were separated on an 1% agarose gel and extracted fragments were cloned into vector pSKpADH1 via *Nde*I and *Hin*dIII restriction sites. Isolated plasmids were analyzed by sequencing.

For linker ligation 2 μg of total RNA were mixed with 2 μg of a phosphorylated DNA oligo nucleotide (Oligo_5P_3ddC) and ligated using T4 RNA ligase (NEB, Frankfurt, Germany) for 1.5 h at 37°C. Column purified reactions were used for cDNA synthesis with Oligo_rev as primer. cDNA of the immunity gene was amplified using the primers Oligo_rev and KlO3rev_NdeI as and then cloned into pSKpADH1 via *Nde*I and *Eco*RV restriction sites. Cloned fragments were analyzed by sequencing.

## Supporting Information

S1 Fig Sequences of the immORFs *PaORF4* (A), *KlORF3* (B) and *DrORF5* (C) with the identified mRNA truncation sites (arrows). Each identified cDNA fragment was followed by a stretch of 7–66 adenyl nucleotides (see also Fig 4). Green numbers indicate the number of fragments identified by the 3´-RACE method and red numbers mark mRNA ends identified by the linker ligation method.
(DOCX)

S2 Fig Primary sequences of the wild-type and the codon optimized immORFs *PaORF4/PaORF4ms* (A) and *KlORF3/KlORF3ms* (B). Almost every possible silent mutation was introduced in order to achieve an increased GC-content (from 21% to 45% (A) and from 22% to 54% (B), respectively). Changed nucleotides are shown in blue.
(DOCX)

S3 Fig Alignment of deduced proteins of *PaORF4* (ORF4), *DrORF5* (ORF5) and NUPAV Piso0_001880 (ORF-1). The deduced sequence of ORF-1 includes an N-terminal extension of the ORF and lacks an ATG.
(DOCX)

S1 Table Yeast strains used in this study. (DOCX)

S2 Table Plasmids used in this study. (DOCX)

S3 Table Oligos used in this study. (DOCX)
