# Supplementary figures and images for: Autoselection of Cytoplasmic Yeast Virus Like Elements Encoding Toxin/Antitoxin Systems Involves a Nuclear Barrier for Immunity Gene Expression
Source: PLoS Genet. 2015 May 14;11(5):e1005005. doi: 10.1371/journal.pgen.1005005 (PMC4431711; doi:10.1371/journal.pgen.1005005)

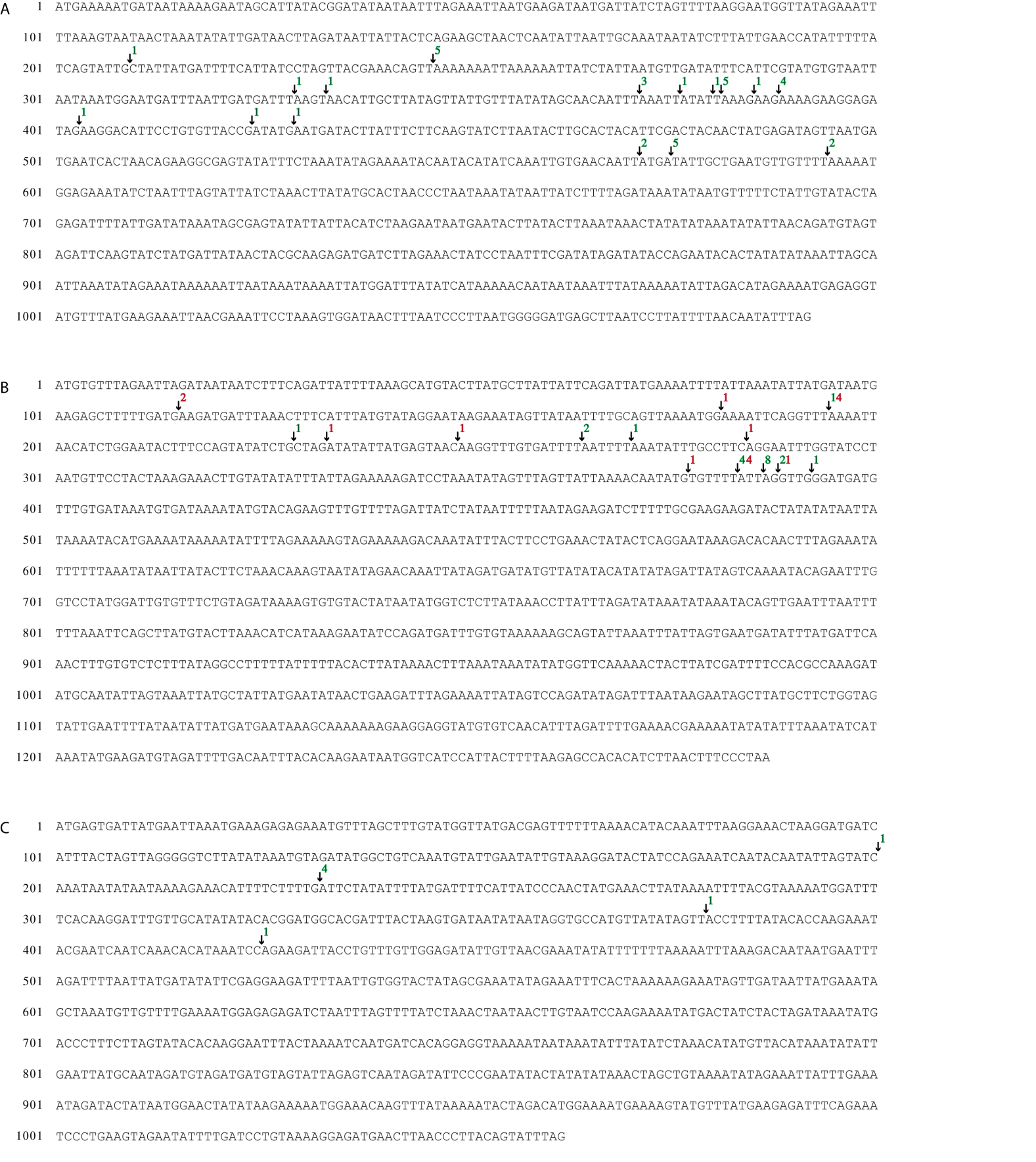

Supplement: S1 Fig — Each identified cDNA fragment was followed by a stretch of 7–66 adenyl nucleotides (see also Fig 4). Green numbers indicate the number of fragments identified by the 3´-RACE method and red numbers mark mRNA ends identified by the linker ligation method. (DOCX) [file pgen.1005005.s001.docx]

A


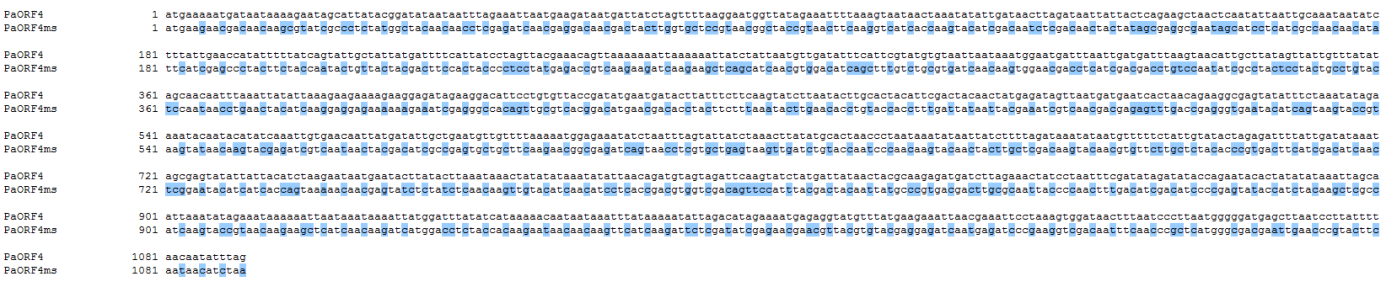


B


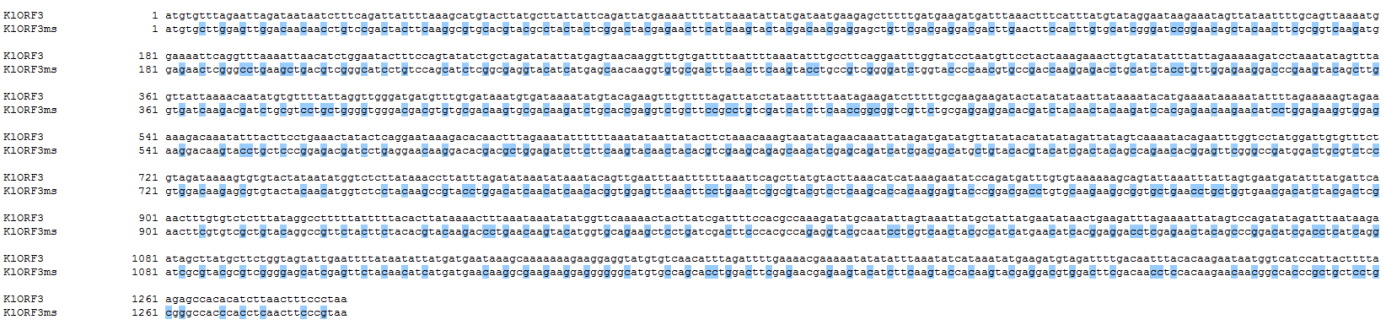

Supplement: S2 Fig — Almost every possible silent mutation was introduced in order to achieve an increased GC-content (from 21% to 45% (A) and from 22% to 54% (B), respectively). Changed nucleotides are shown in blue. (DOCX) [file pgen.1005005.s002.docx]
