# Supplementary material for: Autoselection of Cytoplasmic Yeast Virus Like Elements Encoding Toxin/Antitoxin Systems Involves a Nuclear Barrier for Immunity Gene Expression
Source: PLoS Genet. 2015 May 14;11(5):e1005005. doi: 10.1371/journal.pgen.1005005 (PMC4431711; doi:10.1371/journal.pgen.1005005)
Supplement: S3 Fig — The deduced sequence of ORF-1 includes an N-terminal extension of the ORF and lacks an ATG. (DOCX) [file pgen.1005005.s003.docx]

ORF-1 --------------------------TKILRDVQLIIRGCYLNSNYAKKWVNVLDE--SD

ORF4 -----MKNDNKRIALYGYNNLEI-----NEDNDYLVLRNGYRNFKVITKYIDNLDNYYSE

ORF5 MSDYELNEREKCLALYGYDEFFKTYKFKETKDDHLLVRGSYINVDMAVKCIEYCKGYYPE

: *::* * * . * :: . :

ORF-1 LYTILLANNLYFG--EFYDYFYDFNYPNYETCKLLKQKEY--NIDFIAYFKGWHDLIDYN

ORF4 ANSILIANNIFIEPYFYQYCYYDFHYPSYETVKKIKKLSINVDISFVCVINKWNDLIDDL

ORF5 INTILVSNNIIKETFSFDSIFYDFHYPNYETYKILRKNGF--SQGFVAYIHGWHDLLSDN

:**::**: : :***.**.*** * ::: . *:. :. *.**:.

ORF-1 NPSAMLISYGMYTKNKDLLKNYNPDN-YLDSN-EINETYFMKNLNTRMNYDTLYGIEEYD

ORF4 --SNIAYSYCLYSNNLNYIKEEKKEIEGHSCVTDMNDTYFFKYLNTCTTFDYNYEIVNDE

ORF5 IIGAMLYSYLLYTKKYESIKHINPED-YLFVGDIVNEIYFFKNLKTIMNLDFNYDIFEED

. : ** :*::: : :*. : : :*: **:* *:* . * * * : :

ORF-1 --YECETIVKYRKFSRKEIVDNYDIAKCCFENGDISNLVISKLLACNPDKYSYLFGKYKP

ORF4 SLTEGEYISKYRKYNTYQIVNNYDIAECCFKNGEISNLVLSKLICTNPNKYNYLLDKYNV

ORF5 --FNCGTIAKYRNFTKKEIVDNYEIAKCCFENGERSNLVLSKLITCNPRKYDYLLDKYDP

: * ***::. :**:**:**:***:**: ****:***: ** **.**: **.

ORF-1 FLIYTRTAPLYKYSTFSDVKILKYSYLNSIYIDILHEAIDILVSENSMRI---HFIETDK

ORF4 FLLYTRDFIDINSEYIITSKNNEYLYLNKLYINILTDVVDSSIYDYNYARDDLRNYPNFD

ORF5 FLVYTRNLLK---SMITGGKNNKYLYLNICYINILNYAIDV------------DDVVLES

**:*** . : * :* *** **:** .:* .

ORF-1 IDIPEYTLYKLAVKYRVKEVEDRIMKLYLENNKKFIKILDMENEKYVYEEISEFPETEFF

ORF4 IDIPEYTIYKLAIKYRNKKLINKIMDLYHKNNNKFIKILDIENERYVYEEINEIPKVDNF

ORF5 IDIPEYTIYKLAVKYRNYLKIDTIMELYKKNGNKFIKILDMENEKYVYEEISEIPEVEYF

*******:****:*** : **.** :* :*******:***:******.*:*:.: *

ORF-1 NPIKGDELNPYSE--

ORF4 NPLMGDELNPYFNNI

ORF5 DPVKGDELNPYSI--

:*: *******
