# Supplementary material for: Autoselection of Cytoplasmic Yeast Virus Like Elements Encoding Toxin/Antitoxin Systems Involves a Nuclear Barrier for Immunity Gene Expression
Source: PLoS Genet. 2015 May 14;11(5):e1005005. doi: 10.1371/journal.pgen.1005005 (PMC4431711; doi:10.1371/journal.pgen.1005005)
Supplement: S1 Table — (DOCX) [file pgen.1005005.s004.docx]

| **Strains** | **Description** | **Reference** |
| --- | --- | --- |
| *Pichia acaciae* NRRL Y-18665 | Wild type, pPac1-1, pPac1-2 | [[1](#_ENREF_1)] |
| *Kluyveromyces lactis* AWJ137 | *leu2*, *trp1*, pGKL1, pGKL2 | [[38](#_ENREF_38)] |
| *Debaryomyces robertsiae* CBS6693 | Wild type, pWR1A, pWR1B | [[39](#_ENREF_39)] |
| *S. cerevisiae* 301 | MATα, *his4*-*519*, *112*, *leu2*-*3*, *can1*, *ura3*, pGKL1, pGKL2 | [[16](#_ENREF_16)] |
| *S. cerevisiae* F102.2 MS1607 | as F102.2, but pGKL1-*orf2-LEU2*-PaORF4* | [[16](#_ENREF_16)] |
| *S. cerevisiae* CEN.PK2-1c | MATa, *ura3*–*52*, *leu2*-*3*,*112*, *his3Δ1*, *trp1*-*289*, *MAL*-*2*–*8c*, *SUC2* | [[40](#_ENREF_40)] |
| *S. cerevisiae* S288C BY4741 | MATa, *his3Δ1*, *leu2Δ0*, *met15Δ0*, *ura3Δ0* | from EUROSCARF |
| *S. cerevisiae* S288C BY4741 *elp3* | MATa, *his3Δ1*, *leu2Δ0*, *met15Δ0*, *ura3Δ0*, *elp3*-*kanMX4* | from EUROSCARF |
