# Supplementary material for: Autoselection of Cytoplasmic Yeast Virus Like Elements Encoding Toxin/Antitoxin Systems Involves a Nuclear Barrier for Immunity Gene Expression
Source: PLoS Genet. 2015 May 14;11(5):e1005005. doi: 10.1371/journal.pgen.1005005 (PMC4431711; doi:10.1371/journal.pgen.1005005)
Supplement: S2 Table — (DOCX) [file pgen.1005005.s005.docx]

| **Plasmids** | **Description** | **Reference** |
| --- | --- | --- |
| YEplac195 | 2µ, *URA3*, Amp^R^, *E. coli* ori | [[41](#_ENREF_41)] |
| YCplac111 | *ARS-CEN*, *LEU2*, Amp^R^, *E. coli* ori | [[41](#_ENREF_41)] |
| pSK- | Amp^R^, *E. coli* ori | Stratagene |
| pSKPaO4 | pSK- with *PaORF4* | This work |
| pSKKlO3 | pSK- with *KlORF3* | This work |
| pSKDrO5 | pSK- with *DrORF5* | This work |
| pSKpADH1 | pSK- with *ADH1pr* | [[42](#_ENREF_42)] |
| YEKlO3 | YEplac195 with *ADH1pr*-*KlORF3* fusion | This work |
| YEPaO4 | YEplac195 with *ADH1pr*-*PaORF4* fusion | This work |
| YEDrO5 | YEplac195 with *ADH1pr*-*DrORF5* fusion | This work |
| YCPaO4 | YCplac111 with *ADH1pr*-*PaORF4* fusion | This work |
| YEPaO4ms | YEplac195 with *ADH1pr*-*PaORF4ms* fusion | This work |
| YEKlO3ms | YEplac195 with *ADH1pr*-*KlORF3ms* fusion | This work |
| pABY1644 | pRS316-*GAL1*pr-γ-toxin-GST-KanMX6 | [[10](#_ENREF_10)] |
| pRK57 | YEplac181-*GAL1*pr-γ-toxin-GST | This work |
