# Supplementary material for: Autoselection of Cytoplasmic Yeast Virus Like Elements Encoding Toxin/Antitoxin Systems Involves a Nuclear Barrier for Immunity Gene Expression
Source: PLoS Genet. 2015 May 14;11(5):e1005005. doi: 10.1371/journal.pgen.1005005 (PMC4431711; doi:10.1371/journal.pgen.1005005)
Supplement: S3 Table — (DOCX) [file pgen.1005005.s006.docx]

| **Primers** | **Sequence (5´-3´)** |
| --- | --- |
| Oligo_5P_3ddC | 5’P-GACATACGTACGACGAGTACTGACCAGCTACGATGCATGAGCGCCTGddC-3’ |
| Oligo_rev | CAGGCGCTCATGCAT |
| PO4-NdeI-rv | TTACATATGAAAAATGATAATAAAAGAATAGCATTATACGG |
| PO4-fw | GACCTTAGTGATGTATCAAAATTGAATGG |
| KlO3rev_NdeI | TTTTAATTACATATGTGTTTAGAATTAGATAATAATC |
| KlO3for | CTTTGATAATTTAAATGAATTTGAGAAAATAAG |
| DrO5rev_NdeI | TAACTCATATGAGTGATTATGAATTAAATGAAAG |
| DrO5for | GTCATCTGTATCTAAACTTAACGGTA |
| Mut_NdeI_for | CAATATATTTATGTAACAGATGTTTAGATATAAAT |
| Mut_NdeI_rev | CTAAACATCTGTTACATAAATATATTGAATTATGCAATAG |
| PaO4pf | ATGAAAAATGATAATAAAAGAATAGC |
| PaO4prT7 | AAAAAATAATACGACTCACTATAGGGACTAAATATTGTTAAAATAA |
| KlO3pf | GAATTAGATAATAATCTTTC |
| KlO3prT7 | AAAAAATAATACGACTCACTATAGGGATTAGGGAAAGTTAAGATGTG |
| DrO5pf | GAGTGATTATGAATTAAATG |
| DrO5prT7 | AAAAAATAATACGACTCACTATAGGGACTAAATACTGTAAGGGTTAAG |
| Q0 | CCAGTGAGCAGAGTGACG |
| Qt22 | CCAGTGAGCAGAGTGACGAGGACTCGAGCTCAAGCTTTTTTTTTTTTTTTTTTTTTT |
| ERG3F | ATGGATTTGGTCTTAGAAGTCGC |
| ERG3R | TCAGTTGTTCTTCTTGGTATTTGGG |
| PaO4_ers2 | TCATCCCCCATTAAGGGATTAAAG |
| KlO3_rs3 | AGATGTGTGGCTCTTAAAAG |
| DrO5_rs3 | CTGTAAGGGTTAAGTTCATC |
| PaO4ms_for | ATGAAGAACGACAACAAGCG |
| PaO4ms_rev | TTAGATGTTATTGAAGTACGGGTTC |
| KlO3ms_for | ATGTGCTTGGAGTTGGACAACAACC |
| KlO3ms_rev | TTACGGGAAGTTGAGGTGGGTGGCC |
| PaO4intr-fw | ATTAAGCTCATCCCCCATTAAG |
| PaO4intr-rv | TAAAAGAATAGCATTATACGG |
